# Supplementary material for: Cloning antibodies from single cells in pooled sequence libraries by selective PCR
Source: PLoS One. 2020 Aug 5;15(8):e0236477. doi: 10.1371/journal.pone.0236477 (PMC7406036; doi:10.1371/journal.pone.0236477)
Supplement: S1 Raw images — (PDF) [file pone.0236477.s002.pdf]

S1A Original image of gel shown in Figure 3B

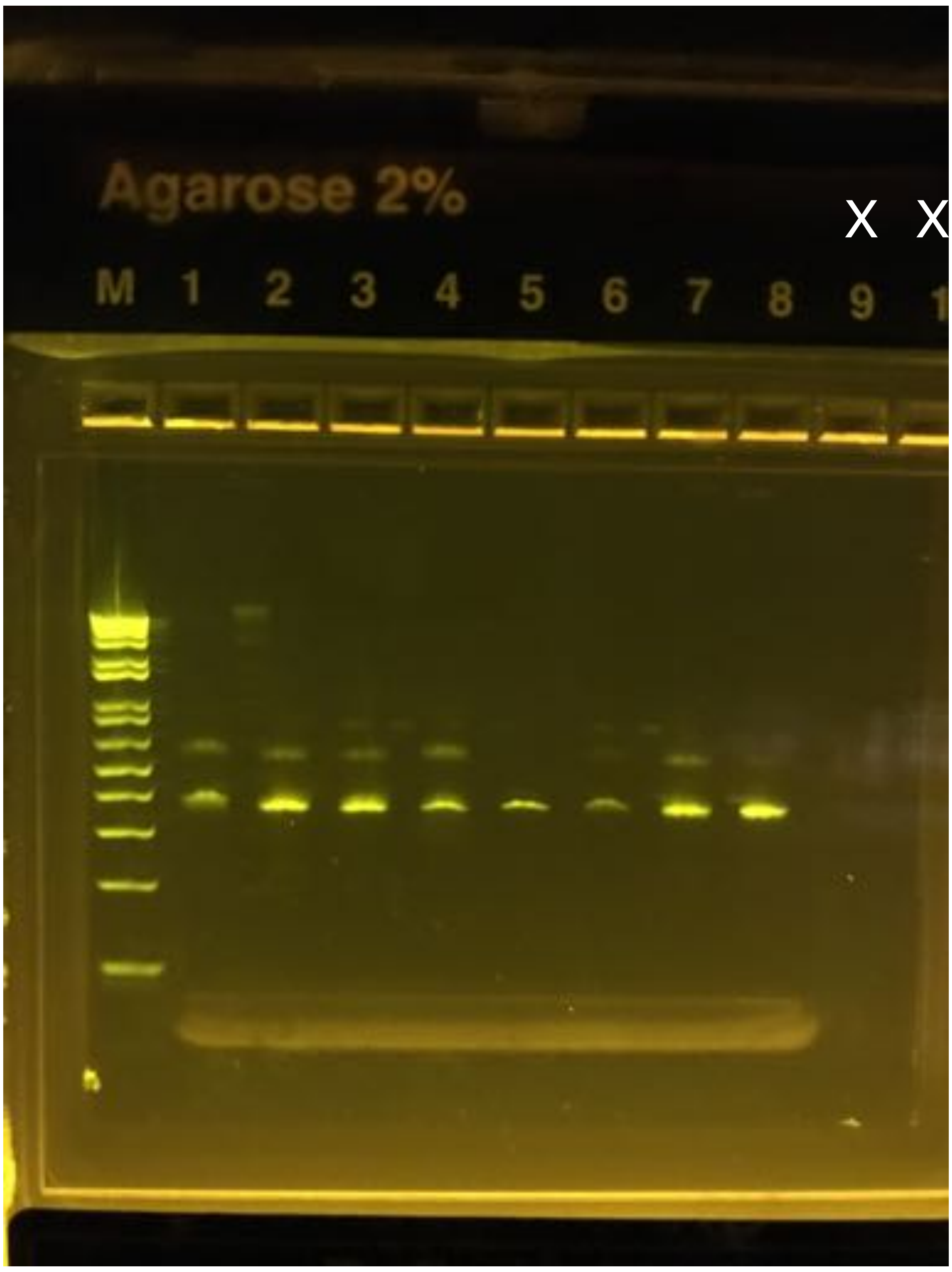

SPAR PCR2 products for heavy-chain genes were separated by electrophoresis on 2% agarose gel. Lanes were loaded from left to right with 1 kb Plus DNA Ladder (ThermFisher), then SPAR PCR2 products for heavy-chain genes of cells 1-8. Lanes 9 and 10 were empty. Image was captured using iPhone 7 camera. Figure 3B was generated from this image by cropping the edges (no adjustment of contrast was made).

S1B Original image of gel shown in Figure 3C

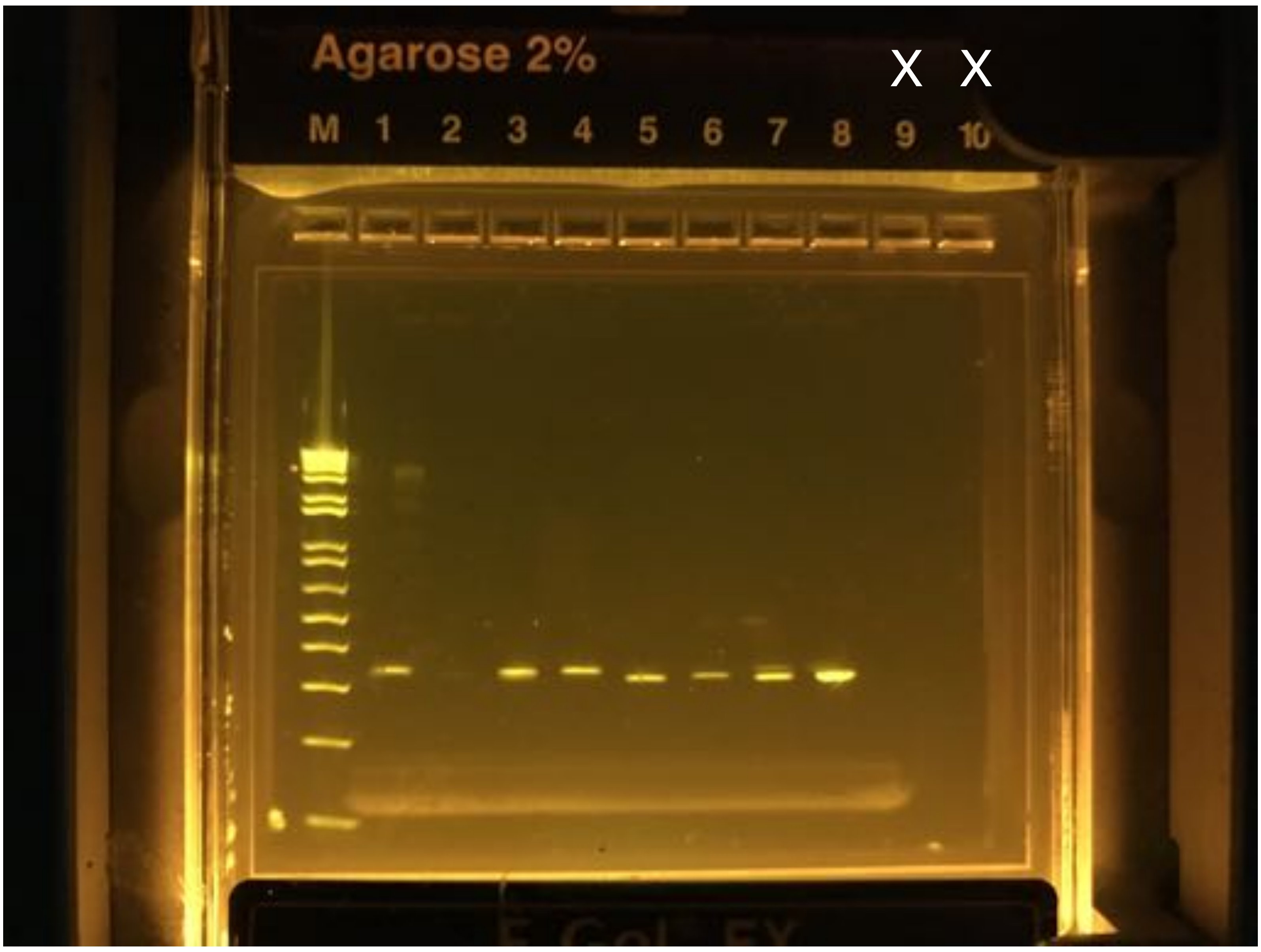

SPAR PCR2 products for light-chain genes were separated by electrophoresis on 2% agarose gel. Lanes were loaded from left to right with 1 kb Plus DNA Ladder (ThermFisher), then SPAR PCR2 products for light-chain genes of cells 1-8. Lanes 9 and 10 were empty. Image was captured using iPhone 7 camera. Figure 3C was generated from this image by cropping the edges (no adjustment of contrast was made).
