# Supplementary material for: Cloning antibodies from single cells in pooled sequence libraries by selective PCR
Source: PLoS One. 2020 Aug 5;15(8):e0236477. doi: 10.1371/journal.pone.0236477 (PMC7406036; doi:10.1371/journal.pone.0236477)
Supplement: Attachment — Submitted filename: Rebuttal_200613.docx [file pone.0236477.s003.docx]

Dear Dr. Henry,

We thank you and the reviewers for carefully reading our paper and identifying these issues. We believe we have now addressed all of them in the revised submission. Please see our point-by-point responses below. Please let us know if we should make any further changes or provide any additional information.

Sincerely,

Felix

**Response to editor comments**

*1. Please ensure that your manuscript meets PLOS ONE's style requirements, including those for file naming.*

We have revised the manuscript to conform to the style requirements.

*2. PLOS ONE now requires that authors provide the original uncropped and unadjusted images underlying all blot or gel results reported in a submission’s figures or Supporting Information files. This policy and the journal’s other requirements for blot/gel reporting and figure preparation are described in detail at [https://journals.plos.org/plosone/s/figures#loc-blot-and-gel-reporting-requirements](https://journals.plos.org/plosone/s/figures#loc-blot-and-gel-reporting-requirements) and [https://journals.plos.org/plosone/s/figures#loc-preparing-figures-from-image-files](https://journals.plos.org/plosone/s/figures#loc-preparing-figures-from-image-files). When you submit your revised manuscript, please ensure that your figures adhere fully to these guidelines and provide the original underlying images for all blot or gel data reported in your submission. See the following link for instructions on providing the original image data: [https://journals.plos.org/plosone/s/figures#loc-original-images-for-blots-and-gels](https://journals.plos.org/plosone/s/figures#loc-original-images-for-blots-and-gels).*

*In your cover letter, please note whether your blot/gel image data are in Supporting Information or posted at a public data repository, provide the repository URL if relevant, and provide specific details as to which raw blot/gel images, if any, are not available. Email us at [plosone@plos.org](mailto:plosone@plos.org) if you have any questions.*

All original gel images are now included as S1_raw_images. These images were used to generate Figure 3B and Figure 3C. We are providing this image data as Supplementary Information. We confirm that the gels reported in the main figures were prepared according to the guidelines. Specifically, no manipulation of the images was performed except for cropping empty lanes.

*3. We note that you have a patent relating to material pertinent to this article. Please provide an amended statement of Competing Interests to declare this patent (with details including name and number), along with any other relevant declarations relating to employment, consultancy, patents, products in development or modified products etc. Please confirm that this does not alter your adherence to all PLOS ONE policies on sharing data and materials, as detailed online in our guide for authors http://journals.plos.org/plosone/s/competing-interests by including the following statement: "This does not alter our adherence to  PLOS ONE policies on sharing data and materials.” If there are restrictions on sharing of data and/or materials, please state these. Please note that we cannot proceed with consideration of your article until this information has been declared.*

*This information should be included in your cover letter; we will change the online submission form on your behalf.*

*Please know it is PLOS ONE policy for corresponding authors to declare, on behalf of all authors, all potential competing interests for the purposes of transparency. PLOS defines a competing interest as anything that interferes with, or could reasonably be perceived as interfering with, the full and objective presentation, peer review, editorial decision-making, or publication of research or non-research articles submitted to one of the journals. Competing interests can be financial or non-financial, professional, or personal. Competing interests can arise in relationship to an organization or another person. Please follow this link to our website for more details on competing interests:* [*http://journals.plos.org/plosone/s/competing-interests*](http://journals.plos.org/plosone/s/competing-interests)

We are preparing to submit a patent application related to this manuscript. We will provide an amended statement of Competing Interests as soon as the application is submitted and the patent name and number are available. This does not alter our adherence to PLOS ONE policies on sharing data and materials, and there are no restrictions on sharing of data or materials. We confirm that all authors do not have potential competing interests.

*4. PLOS requires an ORCID iD for the corresponding author in Editorial Manager on papers submitted after December 6th, 2016. Please ensure that you have an ORCID iD and that it is validated in Editorial Manager.*

We are happy to add the ORCID iDs for all authors. However, when attempting to link the ORCID iD in Editorial Manager, we received a notice “The ORCID site reported the following error and the authorization process cannot be completed:” (The rest of the error message was blank). The ORCID iD of corresponding author Stephen Quake is <https://orcid.org/0000-0002-1613-0809>, and the ORCID iD of author Felix Horns is <https://orcid.org/0000-0001-5872-5061>.

**Response to reviewers**

**Referee 1**

*(1) Did the paired heavy-light chain sequencing cover the whole VH and VL sequences or just some CDRs? If only some CDRs are being sequenced, would this mean that some framework (FR) regions be missed? In particular the FR1, where the primers for PCR2 will bind.*

The paired heavy- and light-chain sequencing covers the entire VH and VL sequences. Thus, no framework regions are missed. We have added a sentence explaining this (Line 100).

*(2) Why can SPAR primers be designed for only 88% of VH and 90% of VL? Can the barcodes misprime to other cDNA sequences? Or is it due to poor sequencing of the framework 1 or the VH or VL?*

For ~10% of heavy- and light-chain genes, SPAR PCR primers satisfying constraints on predicted melting temperature, G/C content, and secondary structure could not be found. While we have considered these genes irretrievable, fine-tuning of PCR conditions might allow them to be retrieved. Additionally, if we used a library preparation method that incorporated a larger CBC and UMI, then there would be a higher likelihood that suitable SPAR PCR1 primers could be designed. We have added a comment on these sources of irretrievability and strategies to mitigate them in the Discussion (Line 183).

*(3) Why was the retrieval and cloning of the VH and VL so inefficient in the end? Figure 3E shows that between 1 and 3 out of 8 clones had 100% DNA sequence identity*

Nearly all of the clones without perfect DNA sequence identity to the target had suffered one or a few single base changes. We attribute this to the imperfect fidelity of the polymerases used for the various PCR steps. This issue could be mitigated by reducing the number of cycles of PCR or using higher fidelity polymerases. We have added a comment about this to the Discussion (Line 189).

**Referee 2**

*The introduction, however, emphasizes that functional evaluation of antibodies is a bottleneck, which is true, but the proposed approach only addresses one piece of the functional screen bottleneck, which is cloning. It's a bit overstated to imply that the proposed approach addresses the functional screen bottleneck.*

We agree that the functional evaluation of antibodies faces further bottlenecks downstream of cloning and expression. We have amended the main text in several places to emphasize that our method addresses the cloning bottleneck (Line 70 in the introduction and Line 162 in the discussion).

*Additionally, while I appreciate the arguments being made, I find tables 1 and 2 as not adding major contributions to the paper. Table 1 for example doesn't include personnel time. The cost of ordering the heavy and light chain genes for synthesis is ~$100, so the cost argument isn't super strong given how easy it is to order genes these days.*

We have clarified that the cost of SPAR is cheaper than or similar in price to gene synthesis (Line 174).

*Second, Table 2 is a bit subjective - would 1 hr of hands on time for primer design be consistent for everyone? In many ways, it is much easier to just order the gene for synthesis. I think there's still value to the approach, however, for anyone who really wants to do an immediate PCR to recover HC+LC genes instead of waiting for gene synthesis, but I don't think this needs to be overhyped.*

We agree that the speed of SPAR is a valuable aspect. We have edited the text to emphasize that the turnaround time is faster than gene synthesis and to highlight the situations where this might be advantageous (Line 176). With respect to Table 2, while it is true that the times are subjective, we believe that it is helpful for practitioners of the method to understand the approximate timelines. This is often presented in molecular biology methods papers. We also believe that these timelines, while approximate, illustrate that the timescale is substantially faster than gene synthesis. Nevertheless, we have now indicated in the Table legend that these times are approximate.
